# Evolution of bone compactness in extant and extinct moles (Talpidae): exploring humeral microstructure in small fossorial mammals

**DOI:** 10.1186/1471-2148-13-55

**Published:** 2013-02-26

**Authors:** Patricia S Meier, Constanze Bickelmann, Torsten M Scheyer, Daisuke Koyabu, Marcelo R Sánchez-Villagra

**Affiliations:** 1 Paläontologisches Institut und Museum, Universität Zürich, Karl Schmid-Strasse 4, Zürich, CH-8006, Switzerland; 2 Current address: Museum für Naturkunde-Leibniz-Institut für Evolutions-und Biodiversitätsforschung, Invalidenstrasse 43, Berlin, D-10115, Germany

**Keywords:** Wolff’s law, Paleohistology, Size, Phylogeny, Placentalia

## Abstract

**Background:**

Talpids include forms with different degree of fossoriality, with major specializations in the humerus in the case of the fully fossorial moles. We studied the humeral microanatomy of eleven extant and eight extinct talpid taxa of different lifestyles and of two non-fossorial outgroups and examined the effects of size and phylogeny. We tested the hypothesis that bone microanatomy is different in highly derived humeri of fossorial taxa than in terrestrial and semi-aquatic ones, likely due to special mechanical strains to which they are exposed to during digging. This study is the first comprehensive examination of histological parameters in an ecologically diverse and small-sized mammalian clade.

**Results:**

No pattern of global bone compactness was found in the humeri of talpids that could be related to biomechanical specialization, phylogeny or size. The transition zone from the medullary cavity to the cortical compacta was larger and the ellipse ratio smaller in fossorial talpids than in non-fossorial talpids. No differences were detected between the two distantly related fossorial clades, Talpini and Scalopini.

**Conclusions:**

At this small size, the overall morphology of the humerus plays a predominant role in absorbing the load, and microanatomical features such as an increase in bone compactness are less important, perhaps due to insufficient gravitational effects. The ellipse ratio of bone compactness shows relatively high intraspecific variation, and therefore predictions from this ratio based on single specimens are invalid.

## Background

Talpidae is a diverse clade of small-sized lipotyphlan mammals which occupy different habitats, ranging from terrestrial to semi-aquatic to fossorial [1]. They are widely distributed throughout the largely temperate regions of the northern continents and their rich fossil record since the Eocene includes many genera from many sites and ages [2]. The phylogenetic relationships within the clade are not yet fully resolved; a comprehensive morphological study [3] and ongoing molecular work [4-6] serve as a framework in which to understand complex biogeographic and ecomorphological patterns of evolution.

**Figure 1:**
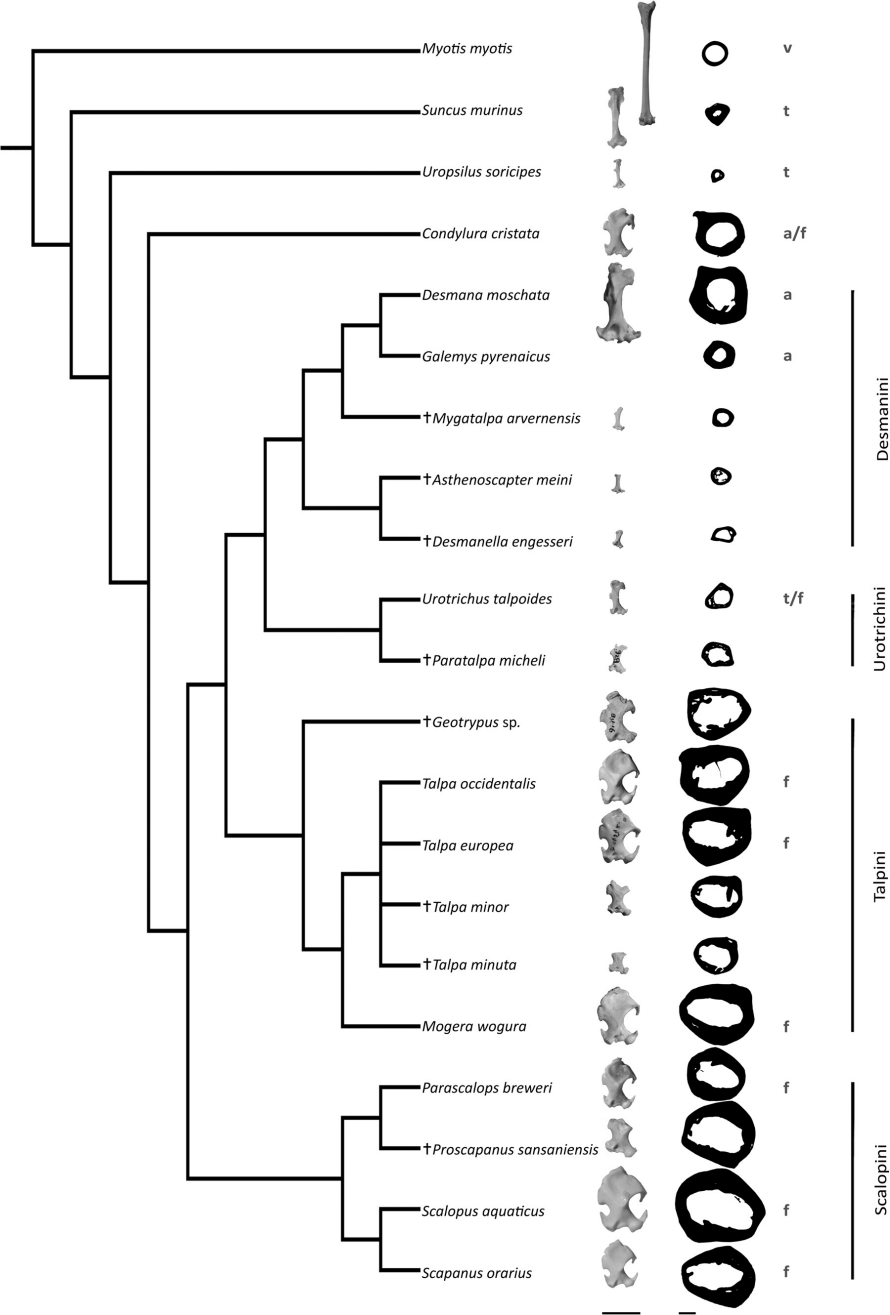
**Humerus (anterior view) and cross sections of each taxon mapped on phylogeny.** Scale bar equals 10 mm for humeri, for cross sections 1 mm. A cross indicates an extinct taxon. The letters refer to the lifestyles, volant (v), terrestrial (t) aquatic (a), and fossorial (f). The cross section of the humerus of *Galemys* was taken from the literature (Laurin et al., 2011).

Talpidae comprise the shrew-like *Uropsilus*, semi-fossorial shrew moles, the Urotrichini, semi-aquatic desmans, the Desmanini, and fossorial moles ([7]; Figure 1). There are two fossorial clades: the Talpini in Eurasia and the Scalopini in North America [1]. The very derived fossorial specializations in morphology are hypothesized to have evolved convergently in these two clades [8]. In the humerus, among other skeletal elements, a transformation from a terrestrial to a fossorial life style occurred, with the most specialized taxa showing a greatly different humeral shape (Figure 1) [9]. Thus, the humerus of fossorial moles is extremely short, broad, and compact, with pronounced muscle attachments [10]. In addition, both the upper and lower bone ends face in opposite direction and this is related to torsion in the mid-shaft region [11]. This humeral morphology is unique among mammals [9,12,13] and seems related to the expansion of muscle attachment sites [14].

The relation between microanatomical structure and mechanical adaptations of long bones, such as the humerus, has been studied in many amniote taxa (e.g., [15-22]). Variations in the histological proportions of cortex and medulla can be biomechanical indicators of lifestyles [17]. In general, terrestrial taxa have a moderately thick, compact cortex with little or no spongiosa in the mid-shaft region, long bones of flying animals show hollow medullas, and terrestrial or swimming taxa have a spongiosa inside the medulla [21]. However, most of these studies focus on adaptations to an aquatic lifestyle [16,19,20,23,24]; in contrast studies of microanatomy in fossorial taxa are lacking.

There is evidence that cortical bone primarily responds to strain only prior to sexual maturity [25,26]. However, Wolff’s ‘Law’, which postulates that bone increases in density and/or cortical thickness in response to the loads it is placed under during an individual’s life, does not always hold, although rules for ‘bone functional adaptation’ to mechanical loading do exist ([27], p. 484; [28,29]). It has been shown that bone thickness is influenced also by other variables such as temperature, and that its development is mediated by genetic mutations and/or modified transcript levels [30-32].

In addition, body size has been suggested to restrict Wolff’s ‘Law’, with bone not responding to biomechanical strains in femora of small animals such as shrews and bats [15]. A high intraspecific variation in cortical thickness in each tested species suggested that Wolff’s Law is not applicable below a certain body size in mammals [15]. In addition, bone density was not significantly different between terrestrial and semi-aquatic rodents within the size range of moles [16]. Phylogeny may be the most important factor coupled with the organisation of bone compactness in mammalian long bones [16,33].

Moles form an ideal subject of research on the evolution of microanatomical structure due to their biomechanical diversity. A relationship between an aquatic and terrestrial lifestyle and humeral microanatomy has been postulated, but this is based on taxonomically broad studies of amniotes [20]. Here, we investigate bone cortical thickness in fossil and living talpid taxa, representing thus one of the first comprehensive examination of bone compactness in any mammalian clade. We test the hypothesis that compactness is higher in humeri in the most fossorial species due to the severe mechanical strains to which they are exposed to during digging. In testing it, we examine several issues around Wolff’s ‘Law’ and others detailed above.

## Materials and methods

Humeri of 11 extant and 8 fossil talpid species representing terrestrial, semi-aquatic and fossorial forms were studied (Figure 1). Two non-talpid species, the Asian house shrew (*Suncus murinus*, Soricidae), and the mouse-eared bat (*Myotis myotis*, Chiroptera), served as outgroups (Additional file 1).

The phylogenetic framework is a composite that best integrates the current knowledge on extant and fossil taxa, a subject not fully resolved [8]. Relationships among extant species follows [3,6], the latter supported the basal position of *Condylura* (see also [8]). The position of *Geotrypus* is based on the recent and comprehensive analysis based on new fossil data by Schwermann & Martin, 2012 [34]. The position of the following fossil taxa is based on the listed references: *Asthenoscapter*[35,36], *Desmanella*[35], *Mygatalpa*[35,36], *Paratalpa*[35,36] and *Proscapanus*[2].

**Figure 2:**
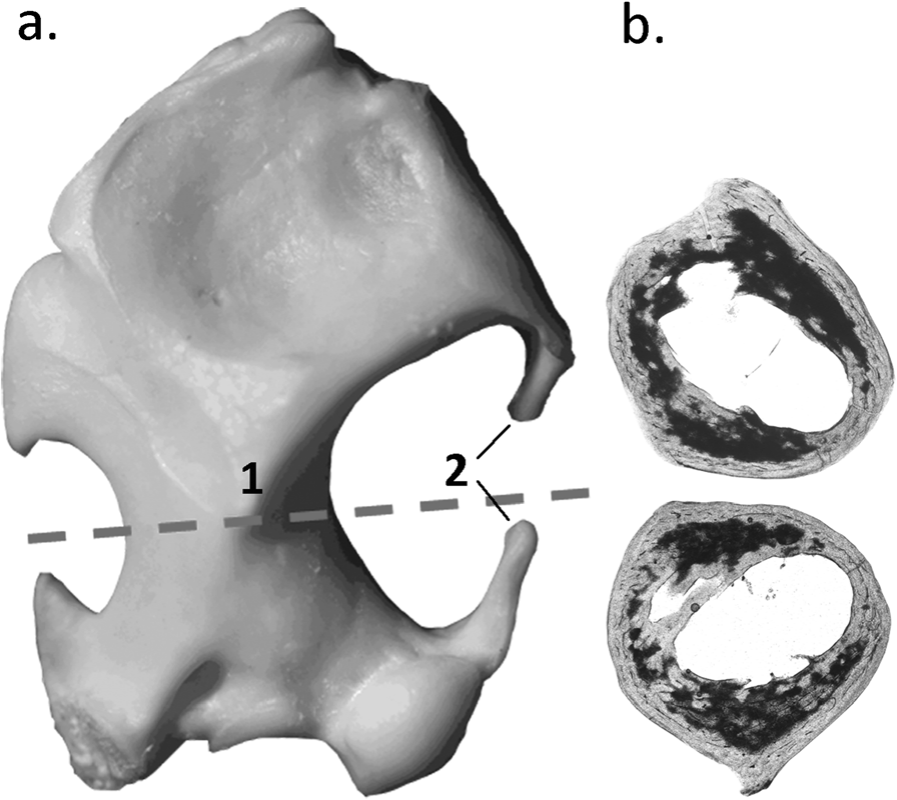
**a. Humerus (anterior view) of *Talpa occidentalis*, dashed line indicates slice plain; (1) distal end of the pectoral crest, (2) elongated tuberculae. b**. the two associated mirroring cross sections, proximal (top) and distal (bottom).

The humerus was sampled because of its abundance and easy recognition in the fossil record as well as its relatively simple bone growth pattern and morphology, with the mid-diaphyseal region yielding the strongest ecological signal (Figure 2) [17,19,20,37]. Bones were photographed prior to preparation following standard petrographic preparation techniques [38,39]. General bone histological features in *Talpa europaea* long bones had been briefly described by Enlow & Brown [40] and were not the focus of this study. The cross section of the humerus of *Galemys pyrenaicus* was taken from Laurin & Canoville [21].

Histomorphometric analyses were performed using the image-processing software BONE PROFILER [18], which has been used to determine bone compactness in amniote long bones [20,24]; for examples of applications see also Houssaye [24] and Hayashi et al. [41]. For whole-cross-section-profiling the program sets a section centre and places a grid over the section dividing the bone tissue into 60 radial sectors and 51 concentric shapes. It then measures the degree of solid bone in each of the 51 subdivisions of each sector. The measurements of all sectors are then integrated into a global compactness of the section. The parameters S (reciprocal of the slope at the inflexion point), P (distance to transition point), Min (lower asymptote of sigmoid curve), Max (upper asymptote), Cc (compactness in the bone centre), CDI (cortico-diaphyseal index; [42]), and Cg (bone global compactness) are calculated by the program. Parameters S, P, and Min have been shown to evince biomechanical information [20].

**Figure 3:**
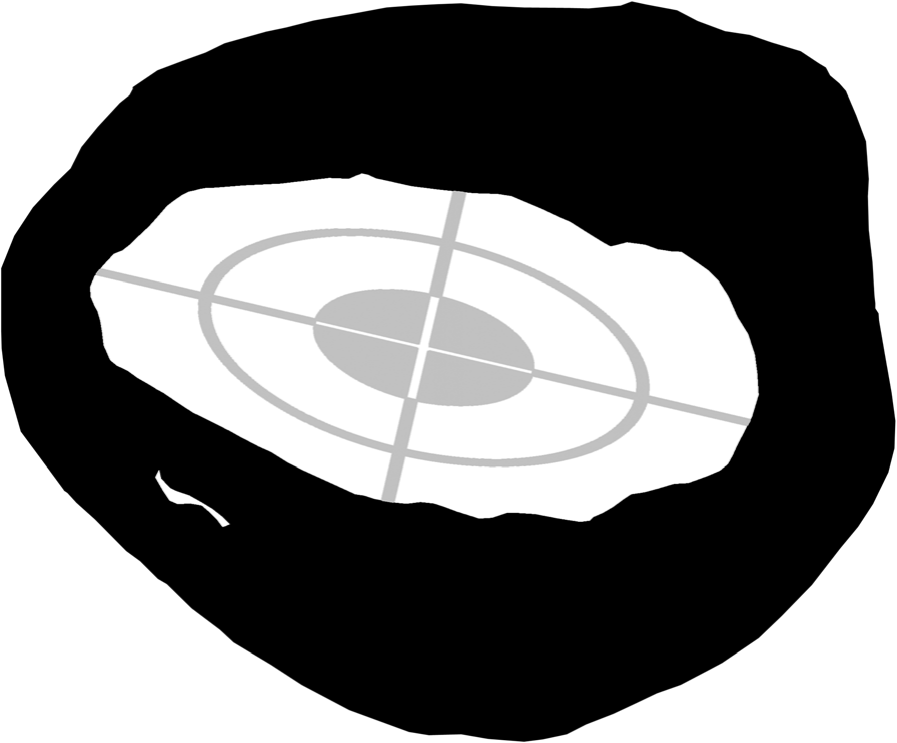
**Measurement of ellipse axes in the medullary cavity of humeral sections.** The long axis of the grid is positioned manually; the short axis is automatically set. The ellipse depicted has no other function as to standardise measurements.

To compare the inner shapes of the cross-sections, the long axis of a standard ellipse was fitted to the medullary cavity of each section. The small axis was set automatically, perpendicular to the long axis (Figure 3). The medullary cavity was chosen for measurements because it is more consistent in its shape than the external bone outline; this can vary depending on the exact location of the section.

Student’s paired t-tests were performed for five bone profile variables (i.e. Cg, CDI, P, S). The groups tested for differences in these values were fully fossorial versus other moles, i.e. Scalopini versus Talpini, and outgroups versus Talpidae. In addition, phylogenetic ANOVA was performed to make phylogenetically corrected between-groups comparisons [43]. We adopted equal lengths for all branches, since estimates of branch lengths for talpids are still very tentative (see [44] for a similar procedure). Phylogenetically corrected contrasts were computed in PDAP module of Mesquite program [43] and assessed by ANOVA in PAST program [45].

## Results

Talpid humeri are hollow in the center, with few showing coarse trabeculae (Figure 1). In general, the humeri of the semi-aquatic Desmanini and terrestrial/semi-fossorial Urotrichini, except for the Russian Desman (*Desmana moschata*), are much smaller than those of the two fossorial clades: Talpini and Scalopini. The inner cross-sections of non-fossorial taxa (Desmanini, *Condylura* and *Urotrichus*) are, overall, more circular compared to those of Talpini and Scalopini. These latter show a rather buckled outline and a slightly elliptic medullary cavity, reflecting the torsion of the humerus and the deep reaching distal end of the deltopectoral crest (Figure 2). The cross-section of *Condylura* is unique in that it displays a typical fossorial outline combined with a very round medullary cavity as present in semi-aquatic and terrestrial species.

The phylogenetic ANOVA analysis (Table 1) shows no significant differences among groups for any of the variables examined (see Additional file 2 for all data). This result could be partly due to the limited numbers of specimens examined, explained after the rarity of available samples for this kind of invasive study. In what follows, we discussed the patterns for each of the variables examined and results of other statistical comparisons.

**Table 1:** Analysis using phylogenetic ANOVA to test for differences on different variables and groups

	**p value**				
**Groups**	**Cg**	**CDI**	**P**	**S**	**Ellipse**
fossorial - non fossorial	0.333	0.367	0.250	0.964	0.141
Scalopini - Talpini	0.618	0.715	0.716	0.741	0.083
Ourgroups - Talpidae	0.477	0.534	0.540	0.555	0.491

### Global compactness (Cg)

**Figure 4:**
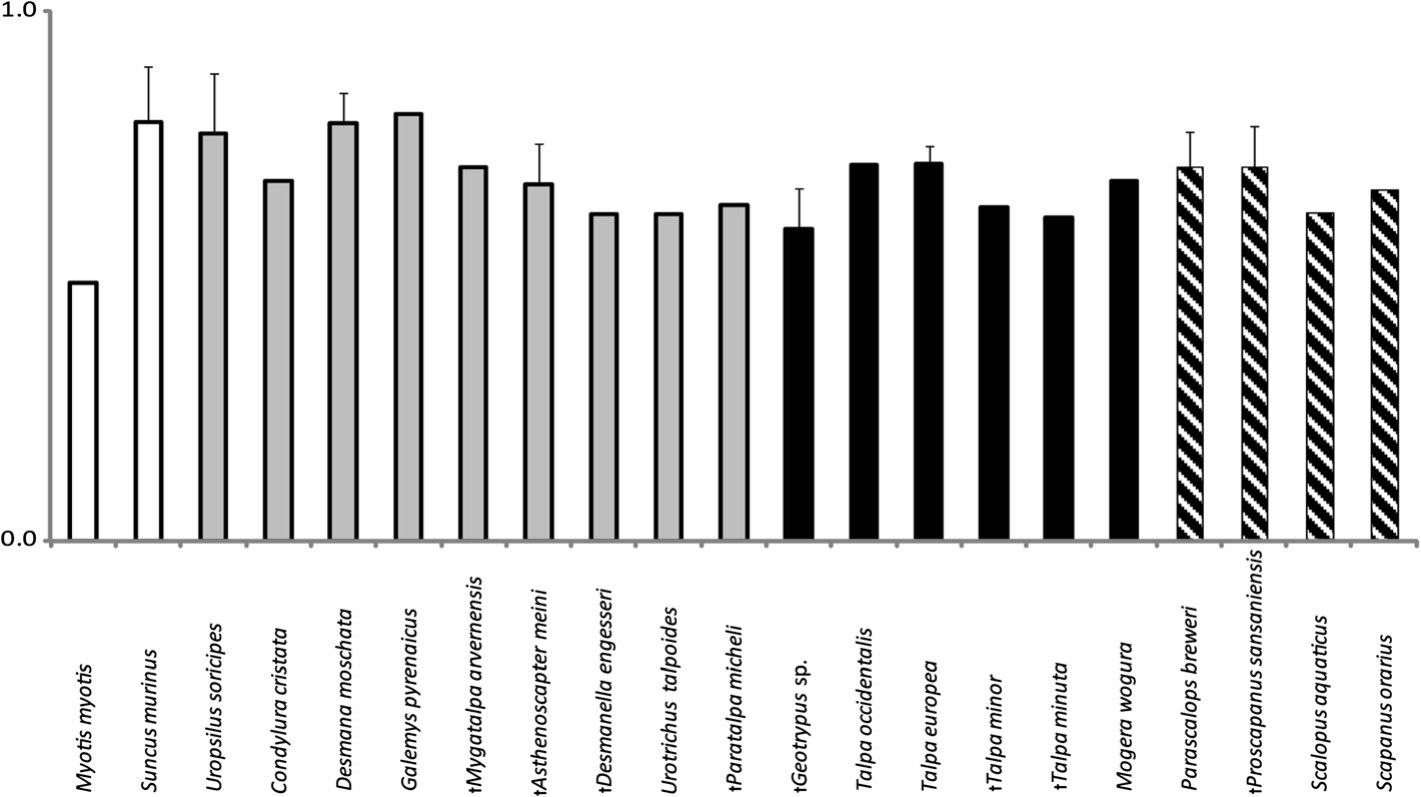
**Means of bone global compactness (Cg) of 18 talpid taxa and 2 outgroups (see Figure**1**).** Standard deviations were plotted for means of taxa represented by at least three cross sections. Outgroup taxa are shown with a white bar; black bars indicate taxa of the Talpini, shaded those of the Scalopini and grey all others.

The global compactness of the bone cross-section (Cg) was more or less equal in all mole taxa and in the shrew, with only the bat having a less compact humerus. Unexpectedly, extinct taxa show an overall slightly lower bone global compactness than most of the extant ones (Figure 4).

### Reciprocal of the slope at the inflexion point (S)

**Figure 5:**
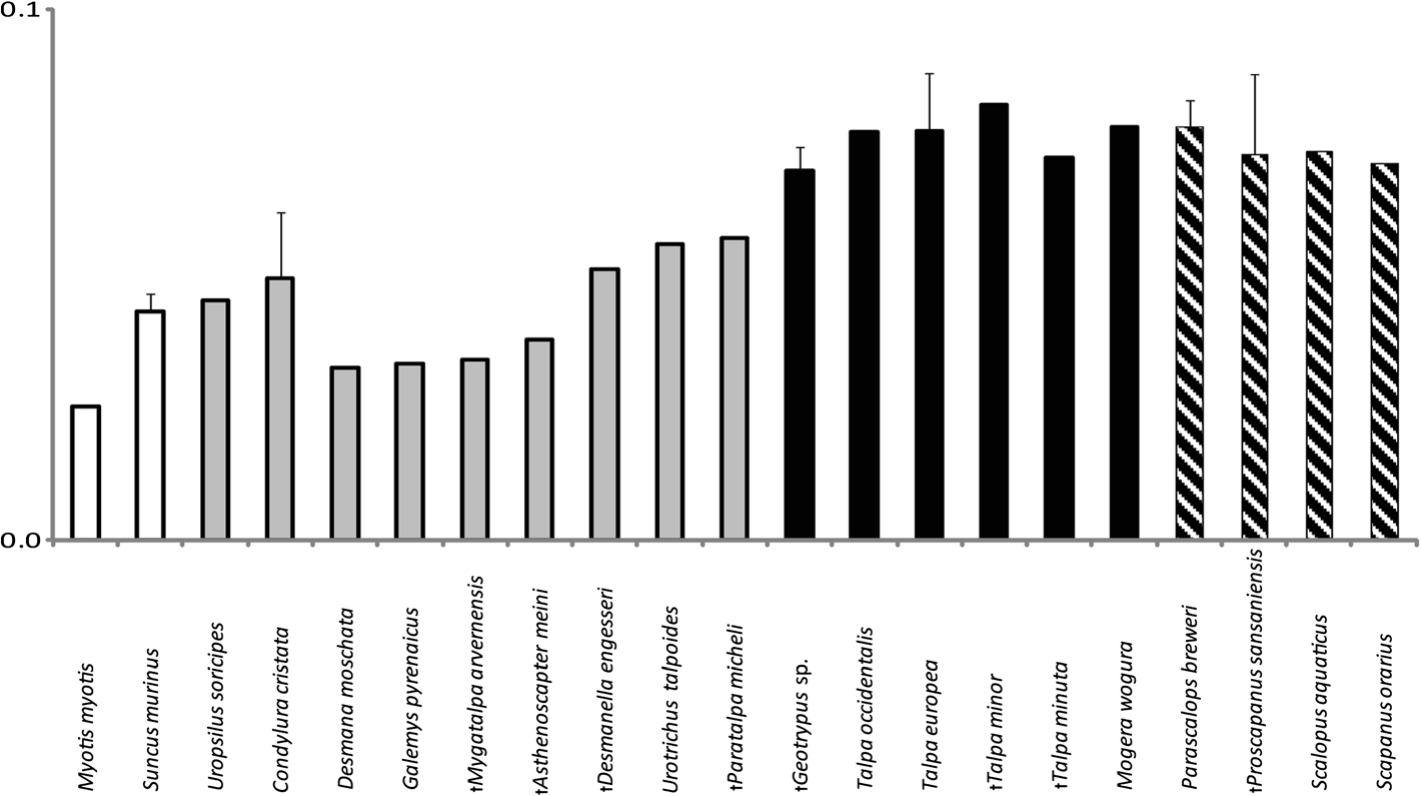
**Means of the compactness parameter S, transition zone of 18 talpid taxa and 2 outgroups (see Figure**1**).** Standard deviations were plotted for means of taxa represented by at least three measurements. Outgroup taxa are shown with a white bar; black bars indicate taxa of the Talpini, shaded those of the tribe Scalopini and grey all others.

S, which reflects the width of the transition zone from the medullary cavity to the cortical compacta, displayed the strongest lifestyle signal in talpid humeri cross-sections. S was significantly higher in fully fossorial Talpidae (0.075 ± 0.005) than in non-fully fossorial ones (0.037 ± 0.009; p < 0.0001) and also in outgroups (0.027 ± 0.013) compared to Talpidae (0.056 ± 0.008; p < 0.05) (Figure 5, Table 2).

**Table 2:** Significance of difference between mean values of the functional groups with Two-sided Student’s t-test, same variance

**a.**	**Fossorial**				**Non fossorial**					
	**Mean**	**+/−**	**n**	**STDEV**	**Mean**	**+/−**	**n**	**STDEV**	**p**	
Cg	0.674	0.038	9	0.059	0.717	0.058	8	0.083	**0.2376**	
CDI	0.451	0.034	9	0.052	0.483	0.055	8	0.079	**0.3338**	
P	0.549	0.034	9	0.052	0.528	0.045	8	0.065	**0.4694**	
S	0.075	0.005	9	0.007	0.037	0.009	8	0.013	**0.0000**	***
ellipse	0.532	0.029	9	0.045	0.782	0.085	8	0.122	**0.0000**	***
**b.**	**Scalopini**				**Talpini**					
	mean	+/−	n	STDEV	mean	+/−	n	STDEV	**p**	
Cg	0.684	0.049	6	0.062	0.644	0.045	9	0.069	**0.2656**	
CDI	0.459	0.049	6	0.062	0.424	0.038	9	0.059	**0.2835**	
P	0.541	0.048	6	0.060	0.576	0.038	9	0.059	**0.2836**	
S	0.071	0.004	6	0.005	0.074	0.005	9	0.008	**0.4144**	
ellipse	0.516	0.022	6	0.028	0.560	0.040	9	0.062	**0.1263**	
**c.**	**Outgroups**				**Talpidae**					
	mean	+/−	n	STDEV	mean	+/−	n	STDEV	**p**	
Cg	0.689	0.221	3	0.195	0.676	0.027	28	0.072	**0.8060**	
CDI	0.451	0.197	3	0.174	0.446	0.024	28	0.065	**0.9205**	
P	0.550	0.197	3	0.174	0.557	0.022	28	0.060	**0.8646**	
S	0.027	0.013	3	0.012	0.056	0.008	28	0.021	**0.0299**	*
ellipse	0.719	0.209	3	0.185	0.648	0.054	28	0.146	**0.4404**	

The asterisks indicate statistical significance (* p < 0.05, *** p < 0.001).

### Ellipse ratio

**Figure 6:**
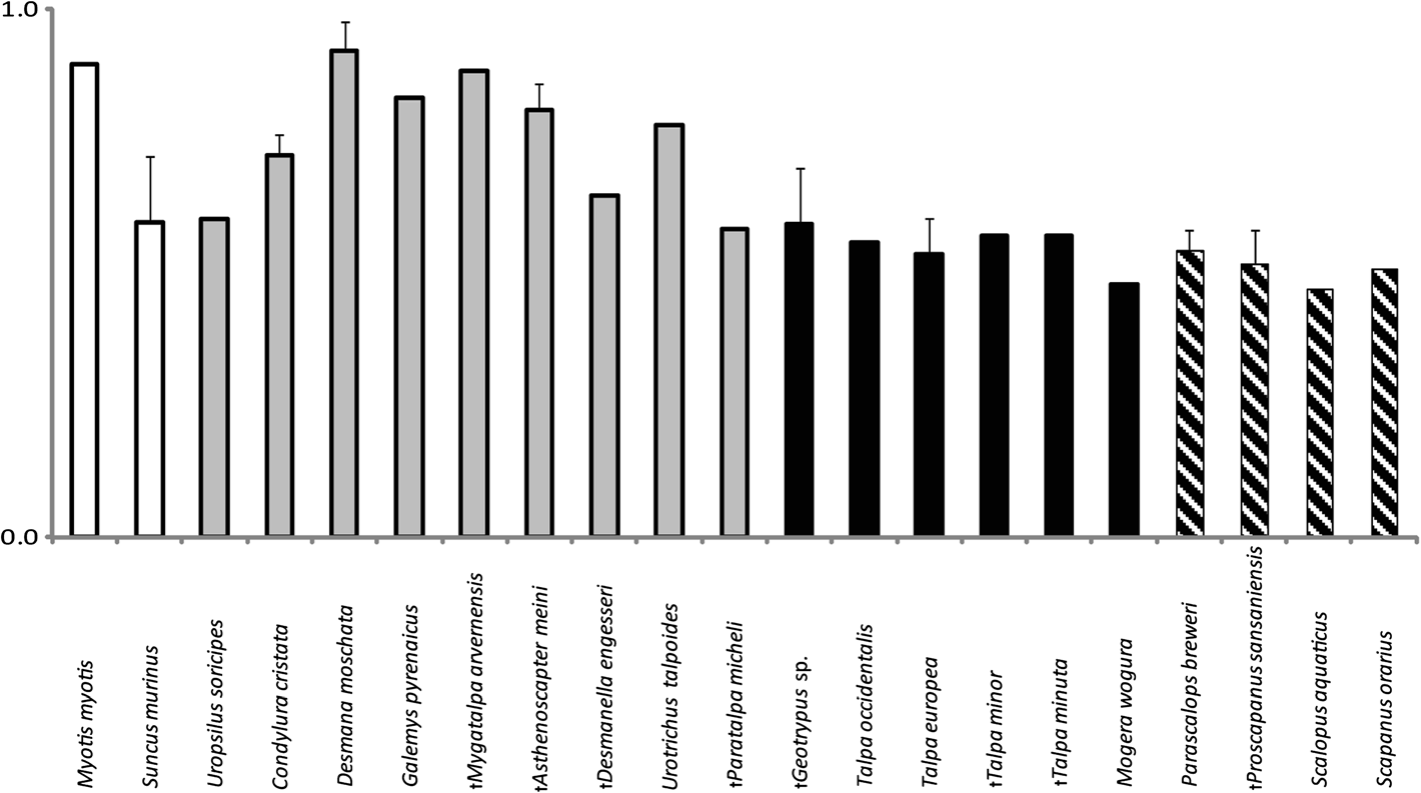
**Ellipse ratios in 18 talpid species and 2 outgroups.** Standard deviations were plotted for means of taxa represented by at least three cross sections. Outgroup taxa are shown with a white bar; black bars indicate taxa of the Talpini, shaded those of the Scalopini and grey all others.

The ellipse ratio, introduced as a measure of quantification of the distortion of the medulla due to the rotation of the condyles in opposite directions, also proved highly significant concerning lifestyles (Figure 6). The fossorial Scalopini and Talpini show a significantly lower ellipse ratio than Desmanini. The ellipse ratio of the semi-fossorial *Urotrichus*, however, is comparable rather to Desmanini. The fossil specimens of *Paratalpa* and *Desmanella* are closer to the Talpini and Scalopini. Ellipse ratios were significantly smaller (more elliptic) in fully fossorial (0.532 ± 0.029) than in partially fossorial (0.782 ± 0.085; p < 0.0001) talpid taxa (Table 2).

The Talpini and Scalopini show no differences in any of the tested variables; all values are within normal range of variance. However, although the results indicate a slightly lower ellipse ratio in Scalopini (0.516 ± 0.41) compared to Talpini (0.560 ± 0.36), these are not statistically significant.

**Figure 7:**
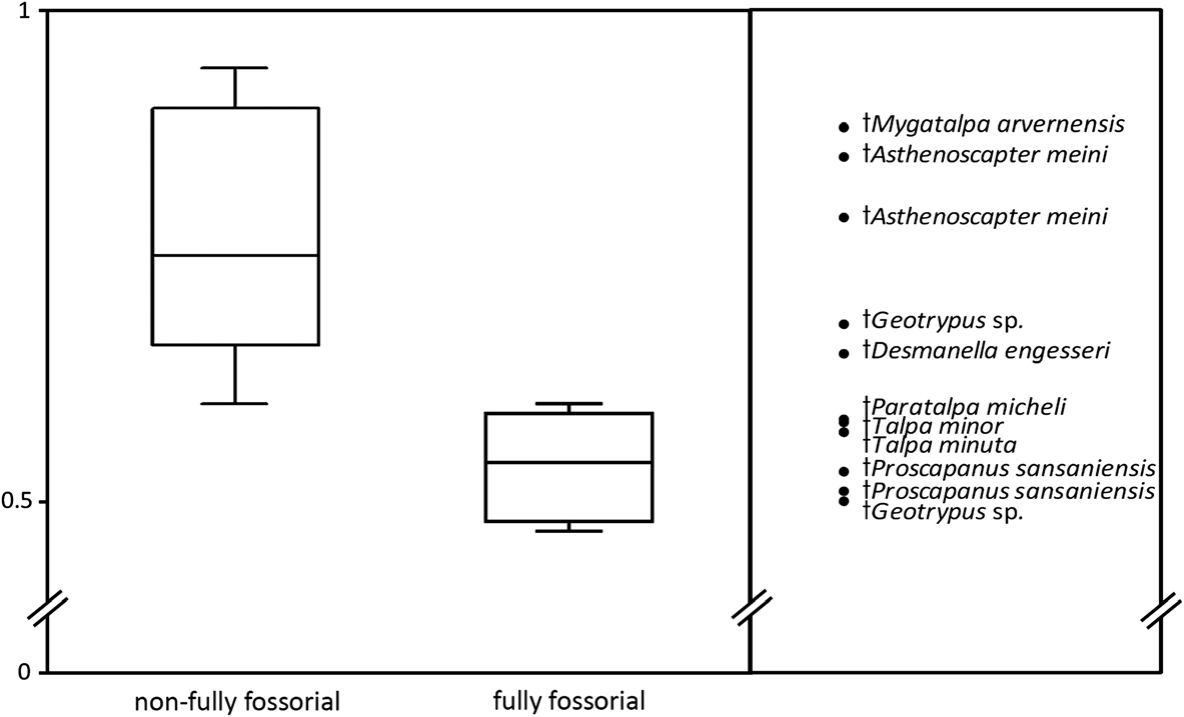
**Ellipse ratios in 18 talpid species and 2 outgroups.** The box plot shows the full distribution of ellipse ratios for non-fully fossorial and fully fossorial talpid taxa: median, quartiles, and extreme values. Boxes represent the interquartile range that contains 50% of values (range from the 25th to the 75th percentile). The line across the box indicates the median. The whiskers represent maximum and minimum values. Extinct taxa were not included in the comparison, but were plotted as separate dots next to the boxes.

The box plot in Figure 7 shows the full distribution of values of the extant taxa in the two compared groups, the fully fossorial species versus other talpids. The fully fossorial and partially fossorial species are well separated. The interquartile ranges are clearly distinct. The extinct species are plotted separately in the same scale in order to visualize membership to either one of the groups. The fossil taxa, *Mygatalpa* and *Asthenoscapter* cluster in the range of non-fossorial extant taxa. This is in accordance with the aquatic lifestyle proposed for these taxa based on habitat reconstruction [35,36]. *Talpa minuta*, *Talpa minor* and *Proscapanus* assort with the fossorial taxa as expected. *Geotrypus* and *Desmanella*, however, do not fall into any specific range.

## Discussion

No difference in global bone compactness (Cg) was detected between the clades of moles or their functional groupings (Figure 4, Tables 1 and 2). It might be expected that Cg would be more elevated in cross-sections of highly derived humeri of fossorial taxa than in terrestrial and semi-aquatic ones in relation to the severe mechanical strains to which they are exposed during digging. The results here reject this hypothesis.

It has been suggested that bone structure in small species is much simpler than in larger ones; for instance, small terrestrial mammals generally have a thin cortex and little or no spongiosa [21]. This implies that the overall morphology of the humerus, at small size, may be all that is required to cope with the strains of digging, and that microanatomical specializations are less likely to occur. In small mammals, the cortical dimensions are probably already mechanically efficient without further adaptation. Dawson [15] calculated the bone tissue strength for the shrew (*Blarina brevicauda*) and two bat species (*Myotis lucifugus* and *Pipistrellus subflavus*), which are comparable in size with moles, using the formula of Koch [46]; the inherent tissue strength (estimated by Ascenzi & Bonucci [47]) exceeded the predicted loading by a factor 100. Dawson [15] thus suggested that Wolff’s Law does not apply in these diminutive mammals. The results from global bone compactness analysis in this study confirm this statement. However, another parameter calculated by BONE PROFILER, the S value, is significantly larger in fossorial talpids than in non-fossorial ones. In amniote long bones in general, S also exhibited an adaptive relationship [20]. In addition, the ellipse ratio showed a highly significant relationship between fossorial and nonfossorial talpids.

No difference was seen in the cross-sections of the two fossorial clades Talpini and Scalopini, which is in congruence with earlier reported results of close convergence in them [8]. Based on stress performance modelling with finite element analysis, Piras et al. ([8], p.13) stated that once the taxa ‘reached the optimal phenotypic status, their humerus did not undergo further morphological changes’. Piras et al. [8] found in the two fully fossorial clades Talpini and Scalopini a slowing of the evolutionary rate of humeri which are better adapted to mechanical stress and the similar path of development of characteristics that lead to a decrease of stress; moreover a lower variance of fossorial humerus shapes when compared to those of non fossorial ones was found.

It has been reported earlier that analysing only one specimen per species can be sufficient as interspecific variation is much higher than intraspecific variation [48]. While this might be true for studies on a higher taxonomic level (e.g. for the Lissamphibia, [48]), intraspecific variation in Talpidae is relatively high, and therefore predictions based on single specimens should be avoided. For example, differences in age, size, sex and nutrition of the specimens can contribute to variation in bone- density [16,49]. An influence on microanatomy by these factors cannot be ruled out. Future studies on bone microstructure variation therefore need not only include additional taxa but also use several specimens per species.

**Figure 8:**
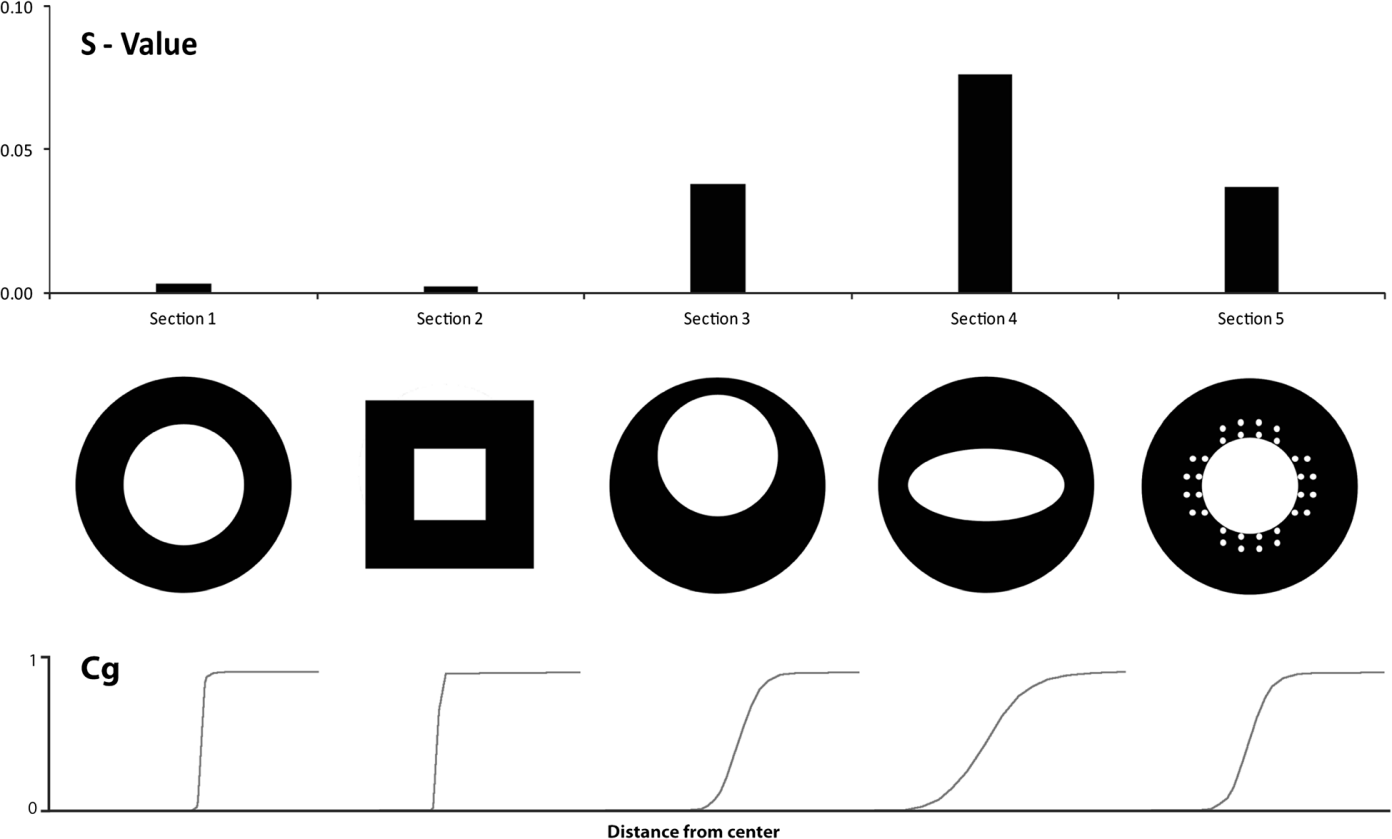
BONE PROFILER analysis of model sections to illustrate different kinds of potential results based on alternative shapes of bones and thus cross sections.

BONE PROFILER is a useful tool for making inferences on the paleobiology of extinct taxa, and thus it is important to understand the performance of the method and the different parameters it produces. In this paper we have shown the influence of outer and inner morphology of the cross-section for inferring lifestyles in several parameters. Figure 8 describes the effect of extreme inner and outer shapes of sections visualized with generated model sections. In talpids, the torsion of the humerus, which is, to this high extent, only present in fully fossorial species, is the reason for the distorted, elliptic medullary cavity, influencing the S-value (Figure 8).

## Conclusions

Fully fossorial talpids are distinguishable from other talpids by the S-value, the reciprocal of slope of the sigmoid curve (Figure 5, Table 2) as well as by the ellipse ratio of the medullary cavity of the humeral cross section (Figure 7), although a phylogenetic corrected statistical analysis did not offer significant results. How these variables behave in other fossorial versus nonfossorial species of mammalian clades of similar size, remains yet to be investigated. Furthermore, the absence of significant differences in the two fully fossorial clades, Talpini and Scalopini, and the low variance compared to nonfossorial taxa, indicate that fossorial adaptation is further evidence of the high degree of evolutionary parallelism in these clades.

## Competing interests

The authors declare that they have no competing interests.

## Authors’ contributions

All authors designed the study. PM, CB and MRSV drafted the manuscript. PM and TMS carried out the histological analyses and PM and DK the statistical analyses. All authors contributed to the manuscript, read it and approved it.

## Supplementary Material

Additional file 1 Specifications on samples. Click here for file

Additional file 2 Bone compactness and variables for the investigated specimens. Click here for file

## References

[B1] Gorman ML Stone DR The natural history of moles 1990 New York: Cornell University Press 138

[B2] Mc Kenna MC Bell SK Classification of mammals above the species level 1997 New York: Columbia University Press

[B3] Sánchez-Villagra MR Horovitz I Motokawa M A comprehensive morphological analysis of talpid moles (Mammalia) phylogenetic relationships Cladistics 2006 22 59 88 10.1111/j.1096-0031.2006.00087.x 34892894

[B4] Shinohara A Campbell KL Suzuki H Molecular phylogenetic relationships of moles, shrew moles, and desmans from the New and Old worlds Mol Phylogenet Evol 2003 27 247 258 10.1016/S1055-7903(02)00416-5 12695089

[B5] Shinohara A Suzuki H Tsuchiya K Zhang Y-P Luo J Jiang X-L Wang Y-X Campbell KL Evolution and biogeography of talpid moles from continental East Asia and the Japanese Islands inferred from mitochondrial and nuclear gene sequences Zoolog Sci 2004 21 1177 1185 10.2108/zsj.21.1177 15613798

[B6] Crumpton N Thompson R The holes of moles: Osteological correlates of the trigeminal nerve in Talpidae J Mamm Evol 2012 10.1007/s10914-012-9213-2

[B7] Koyabu D Endo H Mitgutsch C Suwa G Catania KC Zollikofer CP Oda S-I Koyasu K Ando M Sánchez-Villagra MR Heterochrony and developmental modularity of cranial osteogenesis in lipotyphlan mammals EvoDevo 2011 2 21 10.1186/2041-9139-2-21 22040374 PMC3247175

[B8] Piras P Sansalone G Teresi L Kotsakis T Colangelo P Loy A Testing convergent and parallel adaptations in talpids humeral mechanical performance by means of Geometric Morphometrics and Finite Element Analysis J Morphol 2012 273 696 711 10.1002/jmor.20015 22419178

[B9] Sánchez-Villagra MR Menke PR Geisler JH Patterns of evolutionary transformation in the humerus of moles (Talpidae, Mammalia): a character analysis Nat Hist 2004 170 163 170

[B10] Whidden HP Comparative myology of moles and the phylogeny of the Talpidae (Mammalia, Lipotyphla) Am Mus Novit 2000 3294 1 53

[B11] Freeman R The anatomy of the shoulder and upper arm of the mole (*Talpa europaea*) Journal of Anatomy and Physiology 1886 20 201 219 PMC1288621 17231615

[B12] Reed CA Locomotion and appendicular anatomy in three soricoid insectivores Am Midl Nat 1951 45 513 671 10.2307/2421996

[B13] Yalden DW The anatomy of mole locomotion J Zool 1966 149 55 64

[B14] Gambaryan PP Gasc J-P Renous S Cinefluorographical study of the burrowing movements in the common mole, *Talpa europaea* (Lipotyphla, Talpidae) Russian J Theriol 2002 1 91 109

[B15] Dawson DL Functional interpretations of the radiographic anatomy of the femora of *Myotis lucifugus*, *Pipistrellus subflavus*, and *Blarina brevicauda* Am J Anat 1980 157 1 15 10.1002/aja.1001570102 7405858

[B16] Stein BR Bone density and adaptation in semiaquatic mammals J Mammal 1989 70 467 476 10.2307/1381418

[B17] Francillon-Vieillot H De Buffrénil V Castanet J Géraudie J Meunier FJ Sire J-Y Zylberberg L De Ricqlès AJ Carter JG Microstructure and mineralization of vertebrate skeletal tissues Skeletal biomineralization: Patterns, processes and evolutionary trends 1990 New York: Reinhold, Van Nostrand 471 530

[B18] Girondot M Laurin M Bone Profiler: A tool to quantify, model and statistically compare bone-section compactness profiles Bone 2003 23 458 461

[B19] Germain D Laurin M Microanatomy of the radius and lifestyle in amniotes (Vertebrata, Tetrapoda) Zoologica Scripta 2005 34 335 350 10.1111/j.1463-6409.2005.00198.x

[B20] Canoville A Laurin M Evolution of humeral microanatomy and lifestyle in amniotes, and some comments on palaeobiological inferences Biol J Linn Soc 2010 100 384 406 10.1111/j.1095-8312.2010.01431.x

[B21] Laurin M Canoville A Germain D Bone microanatomy and lifestyle: A descriptive approach Comptes Rendus Palevol 2011 10 381 402 10.1016/j.crpv.2011.02.003

[B22] Hugi J Sánchez-Villagra MR Life history and skeletal adaptations in the Galapagos marine iguana (*Amblyrhynchus cristatus*) as reconstructed with bone histological data—A comparative study of iguanines J Herpetol 2012 46 312 324 10.1670/11-071

[B23] Wall WP The correlation between high limb-bone density and aquatic habits in recent mammals Sediment Geol 1983 57 197 207

[B24] Houssaye A Bone histology of aquatic reptiles: what does it tell us about secondary adaptation to an aquatic life? Biol J Linn Soc 2012 108 3 21

[B25] Pearson OM Lieberman DE The aging of Wolff’s “Law”: Ontogeny and responses to mechanical loading in cortical bone Am J Phys Anthropol 2004 125 63 99 10.1002/ajpa.20155 15605390

[B26] Young MT Brusatte SL Ruta M De Andrade MB The evolution of metriorhynchoidea (Mesoeucrocodylia, Thalattosuchia): An integrated approach using Geometric Morphometrics, Analysis of Disparity, and Biomechanics Zoological Journal of the Linnean Society 2010 158 801 859 10.1111/j.1096-3642.2009.00571.x

[B27] Ruff C Holt B Trinkaus E Who’s afraid of the big bad Wolff?: “Wolff’s law” and bone functional adaptation Am J Phys Anthropol 2006 129 484 498 10.1002/ajpa.20371 16425178

[B28] Frost HM Skeletal structural adaptations to mechanical usage (SATMU): 1. Redefining Wolff’s Law: The bone modeling problem Anat Rec 1990 226 403 413 10.1002/ar.1092260402 2184695

[B29] Frost HM Skeletal structural adaptations to mechanical usage (SATMU): 2. Redefining Wolff’s Law: The remodeling problem Anat Rec 1990 226 414 422 10.1002/ar.1092260403 2184696

[B30] Cretekos CJ Wang Y Green ED Program NCS Martin JF Rasweiler JJ Behringer RR Regulatory divergence modifies limb length between mammals Genes Dev 2008 22 141 151 10.1101/gad.1620408 18198333 PMC2192750

[B31] Serrat MA King D Lovejoy CO Temperature regulates limb length in homeotherms by directly modulating cartilage growth Proc Natl Acad Sci 2008 105 19348 19353 10.1073/pnas.0803319105 19047632 PMC2614764

[B32] Morimoto RI The heat shock response: Systems biology of proteotoxic stress in aging and disease Cold Spring Harb Symp Quant Biol 2011 76 91 99 10.1101/sqb.2012.76.010637 22371371

[B33] Legendre L Le Roy N Martinez-Maza C Montes L Laurin M Cubo J Phylogenetic signal in bone histology of amniotes revisited Zoologica Scripta 2012 42 44 53

[B34] Schwermann A Martin T A partial skeleton of *Geotrypus antiquus* (Talpidae, Mammalia) from the Late Oligocene of the Enspel Fossillagerstätte in Germany Paläontol Z 2012 86 409 439

[B35] Hutchison JH Notes on type specimens of European Miocene talpidae and a tentative classification of Old World Tertiary Talpidae (Insectivora: Mammalia) Geobios 1974 7 211 256 10.1016/S0016-6995(74)80009-4

[B36] Ziegler R Rössner GE, Heissig K Order Insectivora: Talpids The Miocene land mammals of Europe 1999 München: Dr. Friedrich Pfeil 53 74

[B37] Canoville A Laurin M Microanatomical diversity of the humerus and lifestyle in lissamphibians Acta Zoologica 2009 90 110 122 10.1111/j.1463-6395.2008.00328.x

[B38] Kolb C Sánchez-Villagra MR Scheyer TM The palaeohistology of the basal ichthyosaur *Mixosaurus*, Baur, 1887 (Ichthyopterygia, Mixosauridae) from the Middle Triassic: palaeobiological implications Comptes Rendus Palevol 2011 10 403 411 10.1016/j.crpv.2010.10.008

[B39] Chinsamy A Raath MA Preparation of fossil bone for histological examination Palaeontologia africana 1992 29 39 44

[B40] Enlow DH Brown SO A comparative histological study of recent fossil and recent bone tissues. Part III Tex J Sci 1958 10 187 230

[B41] Hayashi S Houssaye A Nakajima Y Chiba K Ando T Sawamura H Inuzuka N Naotomo Kaneko N Tomohiro O Bone histology suggests increasing aquatic adaptations in Desmostylia (Mammalia, Afrotheria) PLoS One 2012 in press 10.1371/journal.pone.0059146 PMC3615000 23565143

[B42] Castanet J Curry Rogers K Cubo J Jacques-Boisard J Periosteal bone growth rates in extant ratites (Ostriche and Emu). Implications for assessing growth in dinosaurs Comptes Rendus de l’Académie des Sciences - Series III - Sciences de la Vie 2000 323 543 550 10.1016/S0764-4469(00)00181-5 10923210

[B43] Garland T Midford PE Ives AR An introduction to phylogenetically based statistical methods, with a new method for confidence intervals on ancestral values Am Zool 1999 39 374 388

[B44] Maximino C Evolutionary changes in the complexity of the tectum of nontetrapods: A cladistic approach PLoS One 2008 3 e3582 10.1371/journal.pone.0003582 18974789 PMC2571994

[B45] Hammer Ø Harper DAT Ryan PD PAST: **Paleontological statistics software package for education and data analysis** Palaeontol Electron 2001 4 9

[B46] Koch JC The laws of bone architecture Am J Anat 1917 21 298

[B47] Ascenzi A Bonucci E The compressive properties of single osteons Anat Rec 1968 161 377 391 10.1002/ar.1091610309 4879362

[B48] Laurin M Girondot M Loth M-M The evolution of long bone microstructure and lifestyle in lissamphibians Paleobiology 2004 30 589 613 10.1666/0094-8373(2004)030<0589:TEOLBM>2.0.CO;2

[B49] Hall BK Bones and cartilage: Developmental and evolutionary skeletal biology 2005 Oxford: Elsevier Ltd 83 90

