# Supplementary material for: Evolution of bone compactness in extant and extinct moles (Talpidae): exploring humeral microstructure in small fossorial mammals
Source: BMC Evol Biol. 2013 Feb 26;13:55. doi: 10.1186/1471-2148-13-55 (PMC3599842; doi:10.1186/1471-2148-13-55)
Supplement: Additional file 1 — Specifications on samples. [file 1471-2148-13-55-S1.pdf]

Appendix 1 Specifications on samples

Taxon	Bone	sin/dext	Continent	Fossil location	Age	PHZ No. [prox/dist] Humerus	Femur	Internal No.	Institute No.	Institute/Donor
Myotis spp.	Humerus	sinistral	Eu			560/561		b1		Zoologisches Institut und Museum Zürich
Suncus murinus	Humerus	sinistral	A			515/516		s1		pers.coll. Koyabu
Suncus murinus	Humerus	sinistral	A			564/-		s2		pers.coll. Koyabu
Condylura cristata	Humerus/Femur	sinistral	Am			511/512	513/514	045		Vanderbilt University Nashville
Condylura cristata	Humerus/Femur	sinistral	Am			507/508	509/510	044		Vanderbilt University Nashville
Condylura cristata	Humerus	sinistral	Am			482/483		032		Vanderbilt University Nashville
Condylura cristata	Humerus	sinistral	Am			458/459		031		Vanderbilt University Nashville
Desmana moschata	Humerus	dextral	EuA			486/ -		036	UMZC E 5292 B	University Museum of Zoology Cambridge
Desmana moschata	Humerus/Femur	dextral	EuA			670/671	672/673	056	-	Paläontologisches Institut und Museum Zürich
Mogera wogura	Humerus	dextral	A			398/399		018	G0064	pers.coll. Sánchez-Villagra
Parascalops breweri	Humerus/Femur	sinistral	Am			505/506	566/-	039		Vanderbilt University Nashville
Parascalops breweri	Humerus	sinistral	Am			484/485		038		Vanderbilt University Nashville
Scalopus aquaticus	Humerus/Femur	sinistral	Am			567/-	568/569	047		pers.coll. Sánchez-Villagra
Scapanus orarius	Humerus/Femur	sinistral	Am			595/-	596/-	053		pers.coll. Sánchez-Villagra
Talpa europea	Humerus/Femur	sinistral	Eu			573/-	574/575	052		pers.coll. Sánchez-Villagra
Talpa europea	Humerus	dextral	Eu			396/397		013	NMB C.1454	Naturhistorisches Museum Basel
Talpa occidentalis	Humerus	sinistral	EuA			401/400		019	T0506	pers.coll. Sánchez-Villagra
Uropsilus soricipes	Humerus/Femur	sin/dext	A			666/667	668/669	055	USNM 574302	Smithsoninan institution, National Museum of Natural History, Washington
Urotrichus talpoides	Humerus	sinistral	EuA			504/-		034	200	Vanderbilt University Nashville
Urotrichus talpoides	Humerus/Femur	sinistral	EuA			- /460	565/-	033	733	Vanderbilt University Nashville
†*Asthenoscapter meini*	Humerus	sinistral	Eu	La Grive		394/-		010	NMB G.a. 3943	Naturhistorisches Museum Basel
†*Asthenoscapter meini*	Humerus	sinistral	Eu	La Grive		664/665		054	NMB G.a.3944	Naturhistorisches Museum Basel
†*Desmanella engesseri*	Humerus	sinistral	Eu	Wintershof-West, Ziegler 1985	lower miocene (MN3)	520/-		027	BSPG 1937 II	Bayrische Staatssammlung für Paläontologie und Geologie München
†*Geotrypus sp.*	Humerus	sinistral	Eu	Branssat		480/481		003	NMB Bst. 16	Naturhistorisches Museum Basel
†*Geotrypus sp.*	Humerus	dextral	Eu	Quercy		386/387		006	NMB QH.434	Naturhistorisches Museum Basel
†*Mygatalpa arvernensis*	Humerus	dextral	Eu	Coderet		392/393		004	(no No.)	Naturhistorisches Museum Basel
†*Paratalpa micheli*	Humerus	dextral	Eu	Coderet		517/-		005	NMB Bst. 9521	Naturhistorisches Museum Basel
†*Proscapanus sansaniensis*	Humerus	sinistral	Eu	Sansan		388/389		007	(no No.)	Naturhistorisches Museum Basel
†*Proscapanus sansaniensis*	Humerus	sinistral	Eu	La Grive	miocene	453/454		015	A/V2793	Paläontologisches Institut und Museum Zürich
†*Talpa europea*	Humerus	sinistral	Eu	Petersbuch 1	mid pleistocene	455/456		023	BSPG 1963 XIV 2	Bayrische Staatssammlung für Paläontologie und Geologie München
†*Talpa minor*	Humerus	sinistral	Eu	Schernfeld, Belege zu Dehm 1962	lower pleistocene	- /457		024	BSPG 1951 XXVI	Bayrische Staatssammlung für Paläontologie und Geologie München
†*Talpa minuta*	Humerus	dextral	Eu	Sansan		391/390		009	(no No.)	Naturhistorisches Museum Basel
