# Supplementary material for: Evolution of bone compactness in extant and extinct moles (Talpidae): exploring humeral microstructure in small fossorial mammals
Source: BMC Evol Biol. 2013 Feb 26;13:55. doi: 10.1186/1471-2148-13-55 (PMC3599842; doi:10.1186/1471-2148-13-55)
Supplement: Additional file 2 — Bone compactness and variables for the investigated specimens. [file 1471-2148-13-55-S2.pdf]

Appendix 2

		Bone Compactness		Average	CDI (Castanet et al. 2000) :		Average	P (=rel. distance from center to inflection point)		Average	S (= reziprocal of slope at inflection point)		Average	Ellipse measurements prox		dist		prox	dist	Average prox dist						
		prox	dist	prox dist	prox	dist	prox dist	prox	dist	prox dist	prox	dist	prox dist	long	short	long	short	ratio	ratio	ratio	extant/ extinct	Body mass g	Body length mm	lifestyle	Tribe	Genus
Myotis spp.	b1 560 561	0.494	0.479	0.487	0.294	0.284	0.289	0.706	0.716	0.711	0.014	0.015	0.014	573	507	587	531	0.885	0.905	0.895	1	26	72	flying		Myotis
Suncus murinus	s1 515 516	0.845	0.908	0.877	0.560	0.710	0.635	0.440	0.290	0.365	0.037	0.037	0.037	407	202	273	152	0.496	0.557	0.527	1	44	126	terrestrial		Suncus
Suncus murinus	s2 564	0.705		0.705	0.428		0.428	0.573		0.573	0.031		0.031	487	358			0.735		0.735	1	44	126	terrestrial		Suncus
Talpa occidentalis	019 401 400	0.705	0.713	0.709	0.474	0.486	0.480	0.526	0.514	0.520	0.071	0.076	0.074	1432	770	1311	752	0.538	0.574	0.556	1	49		fossorial	Talpini	Talpa
Talpa europea	013 396 397	0.716	0.754	0.735	0.483	0.524	0.503	0.517	0.476	0.497	0.064	0.069	0.067	1376	770	1396	641	0.560	0.459	0.509	1	88	130	fossorial	Talpini	Talpa
Talpa europea	023 455 456	0.585	0.542	0.564	0.375	0.343	0.359	0.625	0.657	0.641	0.080	0.087	0.083	1578	847	1527	1004	0.537	0.657	0.597	1	88	130	fossorial	Talpini	Talpa
Talpa europea	052 573	0.690		0.690	0.470		0.470	0.530		0.530	0.088		0.088	1240	728			0.587		0.587	1	88	130	fossorial	Talpini	Talpa
Mogera wogura	018 398 399	0.670	0.687	0.679	0.442	0.466	0.454	0.558	0.534	0.546	0.068	0.081	0.075	1656	791	1547	733	0.478	0.474	0.476	1	97	150	fossorial	Talpini	Mogra
Parascalops breweri	038 484 485	0.676	0.634	0.655	0.449	0.417	0.433	0.551	0.583	0.567	0.070	0.080	0.075	1216	696	1333	677	0.572	0.508	0.540	1	51	127	fossorial	Scalopini	Parascalops
Parascalops breweri	039 505 506	0.788	0.724	0.756	0.557	0.496	0.526	0.443	0.504	0.474	0.070	0.079	0.075	1064	613	1217	626	0.576	0.514	0.545	1	51	127	fossorial	Scalopini	Parascalops
Scalopus aquaticus	047 567	0.620		0.620	0.400		0.400	0.600		0.600	0.069		0.069	2036	956			0.470		0.470	1	87	125	fossorial	Scalopini	Scalopus
Scapanus orarius	053 595	0.663		0.663	0.436		0.436	0.564		0.564	0.067		0.067	1653	839			0.508		0.508	1	62	123	fossorial	Scalopini	Scapanus
Desmana moschata	036 486	0.739		0.739	0.496		0.496	0.504		0.504	0.031		0.031	948	830			0.876		0.876	1	428	203	semiaquatic	Desmanini	Desmana
Desmana moschata	056 670 671	0.824	0.848	0.836	0.587	0.615	0.601	0.587	0.385	0.486	0.018	0.019	0.018	835	747	747	736	0.895	0.985	0.940	1	428	203	semiaquatic	Desmanini	Desmana
Galemys pyrenaicus *	000	0.804		0.804	0.563		0.563	0.437		0.437	0.024		0.024	428	356			0.832		0.832	1	60	125	semiaquatic	Desmanini	Galemys
Condylura cristata	031 458 459	0.676	0.686	0.681	0.439	0.447	0.443	0.561	0.553	0.557	0.044	0.031	0.037	931	680	879	680	0.730	0.774	0.752	1	48	116	semiaquatic/fossorial	Condylurini	Condylura
Condylura cristata	044 57 508	0.677	0.676	0.677	0.447	0.450	0.448	0.553	0.550	0.552	0.061	0.032	0.047	1040	708	909	646	0.681	0.711	0.696	1	48	116	semiaquatic/fossorial	Condylurini	Condylura
Uropsilus soricipes	055 666 667	0.848	0.689	0.769	0.620	0.450	0.535	0.380	0.550	0.465	0.040	0.034	0.037	325	248	680	299	0.763	0.440	0.601	1	16	73	terrestrial		Uropsilus
Urotrichus talpoides	033 460		0.609	0.609		0.389	0.389		0.611	0.611		0.059	0.059			499	448		0.898	0.898	1	18	90	terrestrial	Urotrichini	Urotrichus
Urotrichus talpoides	034 504	0.622		0.622	0.390		0.390	0.610		0.610	0.039		0.039	623	414			0.665		0.665	1	18	90	terrestrial	Urotrichini	Urotrichus
†*Mygatalpa arvernensis*	004 392 393	0.705		0.705		0.462	0.462	0.538		0.538	0.024		0.024			355	313		0.882	0.882	0			unknown		Mygatalpa
†*Asthenoscapter meini*	010 394	0.724		0.724	0.392		0.392	0.608		0.608	0.025		0.025	378	320			0.847		0.847	0			unknown		Asthenoscapter
†*Asthenoscapter meini*	054 664 665	0.573	0.674	0.624	0.353	0.436	0.395	0.647	0.564	0.605	0.028	0.033	0.031	800	659	642	484	0.824	0.754	0.789	0			unknown		Asthenoscapter
†*Desmanella engesseri*	027 520	0.617		0.617	0.390		0.390	0.610		0.610	0.044		0.044	431	278			0.645		0.645	0			unknown		Desmanella
†*Paratalpa micheli*	005 517	0.634		0.634	0.406		0.406	0.594		0.594	0.051		0.051	629	367			0.583		0.583	0			unknown		Paratalpa
†*Geotrypus sp.*	003 480 481	0.677	0.627	0.652	0.449	0.405	0.427	0.551	0.595	0.573	0.068	0.064	0.066	1441	944	1452	1028	0.655	0.708	0.682	0			unknown		Geotrypus
†*Geotrypus sp.*	006 386 387	0.545	0.507	0.526	0.341	0.309	0.325	0.659	0.691	0.675	0.070	0.059	0.064	1373	717	1637	782	0.522	0.478	0.500	0			unknown		Geotrypus
†*Talpa minor*	024 457		0.629	0.629		0.412	0.412		0.588	0.588		0.079	0.079			1120	636		0.568	0.568	0			unknown	Talpini	Talpa
†*Talpa minuta*	009 391 390	0.591	0.629	0.610	0.375	0.408	0.391	0.625	0.592	0.609	0.065	0.071	0.068	985	545	925	542	0.553	0.586	0.570	0			unknown	Talpini	Talpa
†*Proscapanus sansaniensis*	007 388 389	0.676	0.612	0.644	0.449	0.389	0.419	0.551	0.611	0.581	0.070	0.056	0.063	1618	917	1595	780	0.567	0.489	0.528	0			unknown	Scalopini	Proscapanus
†*Proscapanus sansaniensis*	015 453 454	0.774	0.760	0.767	0.559	0.526	0.542	0.442	0.474	0.458	0.093	0.057	0.075	1345	597	1230	702	0.444	0.571	0.507	0			unknown	Scalopini	Proscapanus
